# The Effects of the Combination of Rhein and Platelet-Rich Plasma on Human Articular Chondrocytes

**DOI:** 10.3390/life13081723

**Published:** 2023-08-11

**Authors:** Mario Simental-Mendía, Sonia Amelia Lozano-Sepúlveda, Marsela Garza-Tapia, Jorge Lara-Arias, Carlos Alberto Acosta-Olivo, Félix Vilchez-Cavazos, Víctor Manuel Peña-Martínez

**Affiliations:** 1 Orthopedic Trauma Service, “Dr. José Eleuterio González” University Hospital, Universidad Autónoma de Nuevo León, Monterrey 66455, Mexico; mario.simentalme@uanl.edu.mx (M.S.-M.); jorge.larars@uanl.edu.mx (J.L.-A.); carlos.acostalv@uanl.edu.mx (C.A.A.-O.); jose.vilchezcvz@uanl.edu.mx (F.V.-C.); 2 Department of Biochemistry and Molecular Medicine, School of Medicine, Universidad Autónoma de Nuevo León, Monterrey 66455, Mexico; sonia.lozanosp@uanl.edu.mx; 3 Department of Analytical Chemistry, School of Medicine, Universidad Autónoma de Nuevo León, Monterrey 66455, Mexico; marsela.garzatp@uanl.edu.mx

**Keywords:** chondrocytes, platelet-rich plasma, rhein, knee osteoarthritis

## Abstract

Background: The presence of side effects and low bioavailability of rhein has limited its use in the treatment of osteoarthritis. We aimed to evaluate the in vitro response of human articular chondrocytes to the presence of the combination of platelet-rich plasma (PRP) and rhein. Methods: Solutions of rhein were prepared to assess solubility and select a working concentration. A stimulus with interleukin-1β (IL-β, 10 ng/mL) was induced for 24 h on human chondrocytes. Five treatment groups were established: control, IL-β control, PRP, rhein, and PRP + rhein. Cell viability, cell migration, nitric oxide (NO) production, tumor necrosis factor-α (TNF-α), and gene expression analyses were carried out. Results: A concentration of 50 mg/L was selected after a dose–response curve assay. Both NO and tumor TNF-α production significantly decreased after PRP and PRP + rhein treatments at 24 and 48 h. The wound healing assay revealed a significant stimulation of migration after 72 h with the PRP and PRP + rhein treatments. Expression of IL-1β, IL-6, MMP-13, and ADAMTS-5 was significantly downregulated, particularly after treatment with the combination of PRP + rhein. Conclusions: Much of the determinations denoted a better performance of the combination of PRP and rhein in decreasing the levels of the different targets evaluated; however, this was not great enough to detect a significant difference in comparison with the PRP treatment alone.

## 1. Introduction

Osteoarthritis (OA) is a chronic degenerative disorder mostly characterized by joint pain and stiffness which leads to a progressive decline in joint function and a deterioration in quality of life [1,2]. The prevalence of OA increases with age, with an estimated almost twice as many cases in women than in men over 60 years of age [3]. Particularly, obesity (BMI ≥ 30 kg/m^2^) represents an increased risk factor for the development of symptomatic knee OA (19.7% versus 10.9% of nonobese persons) [4]. As OA progresses, the joint undergoes a series of structural changes as part of the pathophysiological process, such as cartilage degradation, bone remodeling, osteophyte formation, and synovial inflammation [5].

Despite the high prevalence of OA worldwide, the therapeutic options initially recommended by the main clinical treatment guidelines continue to be topical and oral non-steroidal anti-inflammatory drugs (NSAIDs) [6]. This occurs in the absence of approved pharmacological agents that may interfere with the progression of the disease.

As an alternative to NSAIDs, other molecules such as glucosamine, chondroitin sulfate, and diacerein have been considered as secondary interventions that may improve or control OA-associated symptomatology [7]. Diacerein, of which the active metabolite is rhein, is an anthraquinone derivative that has been shown to inhibit the production and activity of the proinflammatory cytokine interleukin-1β (IL-1β) [8,9]. Rhein has been also credited with different biological properties, including anti-inflammatory and chondroprotective activities [10]. Diacerein can improve pain and joint function, but its oral administration is associated with gastrointestinal secondary effects [11].

Intra-articular injections of platelet-rich plasma (PRP) have served as an effective emerging therapeutic alternative in the improvement of symptoms caused by OA [12]. This fact has also been supported by the beneficial effects that PRP exerts on certain tissues and cell types within the joint (particularly cartilage and chondrocytes), such as promoting cell proliferation, anti-inflammatory, antiapoptotic, anabolic, and anticatabolic effects [13].

This study aimed to evaluate the response in vitro of human articular chondrocytes in the presence of the combination of PRP and the active metabolite of diacerein (rhein), in search of a therapeutic alternative with a potential synergistic effect.

## 2. Materials and Methods

### 2.1. Patients and Samples

Articular cartilage was obtained from patients undergoing total knee replacement surgery. Tissue handling and procurement of human tissues was approved by the Institutional Research Ethics Committee (approval No. OR 18-00004). Informed consent was obtained from all patients. Cartilage samples were obtained from nine patients (six female and three male) with a median of 70 years and an interquartile range of 61–79 years. Samples from infected joints were excluded, and samples from patients in whom the tissue sample was insufficient or not presenting viable tissue for processing were also eliminated. To recover viable cartilage, the procured joint tissue was cut using an Osteochondral Autograft Transfer System (OATS^®^, Arthrex Inc., Naples, FL, USA). Precise circular samples measuring 6–8 mm in diameter were meticulously extracted. The goal was to obtain cartilage specimens free from erosion or any exposure of the subchondral bone. These samples were specifically taken from non-weight-bearing regions located at the periphery of the femoral condyles.

### 2.2. Isolation and Culture of Articular Chondrocytes

Isolation of articular chondrocytes was carried out through several cycles of enzymatic digestion. Briefly, cartilage was mechanically disrupted into small pieces (1 mm^3^ approximately) using a scalpel, and subsequently subjected to enzymatic breakdown (30 min at 37 °C with constant stirring at 90 rpm) with trypsin without EDTA (GIBCO^®^-BRL Life Technologies, Grand Island, NY, USA). Then, digestion continued with at least two cycles of type II collagenase 2 mg/mL at 37 °C with constant stirring at 90 rpm. Cells contained in the supernatant were pelleted via centrifugation at 1800 rpm for 5 min for each digestion cycle. Chondrocytes were suspended in complete DMEM/F12 (GIBCO^®^-BRL Life Technologies, Grand Island, NY, USA) culture medium supplemented with 10% fetal bovine serum (FBS, GIBCO^®^-BRL Life Technologies, Grand Island, NY, USA) and gentamicin (0.05 mg/mL, Laboratorios Química SON’S, Puebla, Mexico), and transferred to 75 cm^2^ culture flasks at 37 °C in a 5% CO_2_ environment and relative humidity of 100%. Cells with less than three passages were used.

### 2.3. Rhein Solubility Determination

Solubility tests were performed by following the OECD (Organisation for Economic Cooperation and Development) guidelines for the testing of chemicals No. 107 (shake flask method) at a constant temperature [14]. Briefly, a saturated rhein solution was left under constant stirring for 24 h at 25 °C, followed by 24 h of rest at the same temperature, and finally centrifuged at 3500 rpm at 25 °C for 5 min to separate the solution from the undissolved solid. The tests were performed on pooled human PRP samples activated with 10% calcium gluconate in a ratio of 0.15 mL for each mL of serum (to simulate the combination of PRP and rhein if injected in an OA knee). For rhein quantification, a high-performance liquid chromatography (HPLC) method was developed using a Waters 3695 chromatograph with a diode array detector (Waters Corporation, Milford, MA, USA). The developed method was validated considering ICH (International Council for Harmonisation of Technical Requirements for Pharmaceuticals for Human Use) recommendations [15]. The parameters evaluated were linearity, precision, accuracy, limit of detection, and limit of quantification. To validate the method, a 200 µg/mL concentrated solution of rhein was prepared in a mixture of 100 mM ammonium/ammonia buffer (pH 9) and methanol (60:40). Working solutions were prepared in a range of 0.25 to 16 µg/mL [16,17,18,19] in the same mixture of solvents.

### 2.4. Determination of Rhein Working Concentration

Once the rhein solubility in human plasma was determined, we aimed to establish the working concentration of rhein by evaluating the cell viability of cultured human chondrocytes with a dose–response assay (5, 10, 25, 50, 75 mg/L), considering previous data from the literature [16,17,18,19,20]. Chondrocyte viability was evaluated through an ATP quantitation assay of metabolically active cells (CellTiter-Glo^®^ Luminescent Cell Viability Assay, Promega, WI, USA). This assay is ideal for automated high-throughput screening and cell proliferation and cytotoxicity examinations. Chondrocytes were cultivated in 96- opaque-walled plates suitable for luminescence measurements (Thermo Fisher Scientific, Nunc, Denmark) at a cellular density of 2 × 10^5^ cells per well with complete DMEM/F12 for 24 h. Mono-oxygenation of luciferin is catalyzed by luciferase in the presence of Mg^2+^, ATP and molecular oxygen. There is a direct relationship between the luminescent signal and the number of cells in the culture.

### 2.5. Establishment of Experimental Groups

Chondrocytes were cultivated in 24-well plates (Costar^®^ Corning Incorporated, Corning, NY, USA) at a density of 5 × 10^5^ cells/well with complete DMEM/F12 (10% FBS and gentamicin). After the cells were attached, a stimulus with interleukin-1β (IL-β, 10 ng/mL) for 24 h was induced. Then, five treatment groups were established: (1) negative control (chondrocytes without IL-1β stimulus), (2) IL-1β (control of inflammation), (3) PRP (10% PRP), (4) rhein (50 mg/L), and (5) PRP + rhein (10% PRP + 50 mg/L). Samples were obtained from each group at different time points (0–72 h) of culture for total RNA extraction or supernatant culture medium storage at −80 °C for subsequent analysis.

### 2.6. Cell Migration

The migration of chondrocytes was evaluated using a wound healing assay technique by creating a cell-free vertical wound area in the confluent monolayer through mechanical damage using a pipette tip [21,22]. The tip was placed firmly and perpendicular to the surface at 90° to produce a vertical wound. Then, the cell debris was aspirated, and a new culture medium was added. The gap generated was evaluated with microphotographs at 0, 24, 48, and 72 h. The wound healing size tool plugin for ImageJ was used to estimate the area (percentage) devoid of cells through densitometric analysis [23]. The color parameters, distribution, saturation, and luminance were established in the software, and were the same for all the images obtained. The images were transformed to a gray scale and the percentage of the area devoid of chondrocytes was estimated. The images obtained were analyzed with the ImageJ program version 1.49 (National Institutes of Health).

### 2.7. Total RNA Extraction and Gene Expression Analysis

Total RNA was isolated from chondrocytes at the described time intervals and in the described culture conditions with the RNeasy Mini Kit (QIAGEN, Hilden, Germany) following the manufacturer’s specifications. The yield and purity of the RNA were measured spectrophotometrically with NanoDrop 2000 equipment (Thermo Fisher Scientific Inc., Waltham, MA, USA). Complementary DNA (cDNA) synthesis and quantitative polymerase chain reaction (qPCR) reactions were performed using a GoTaq Probe 2-Step RT-qPCR System (Promega Corporation, Madison, WI, USA). Chondrocytes were examined for their gene expression concerning inflammatory response and extracellular matrix (ECM) synthesis. Specific probes were used for each gene (Integrated DNA Technologies, Inc., Coralville, IA, USA), and the evaluation was conducted in triplicate for each tested condition and experimental group. A 7500 Fast Real-Time PCR System (Applied Biosystems; Thermo Fisher Scientific, Inc., Foster City, CA, USA) was utilized for gene expression assays. The list of genes assessed can be found in Table 1. Data analysis involved the ΔΔCq method for relative expression, with β-2-microglobulin (B2M) serving as the endogenous gene for normalization [24].

### 2.8. Quantification of Nitric Oxide Production

The levels of nitric oxide (NO) in the culture supernatants were assessed using the Griess reagent (Promega Corporation, Madison, WI, USA). This reagent converts available nitrate into nitrite with the aid of nitrite reductase. Absorbance at 540 nm was measured in 96-well plates using a Cytation3 HT multimodal plate reader (BioTek Instruments Inc., Winooski, VT, USA). A standard curve was generated using sodium nitrate to determine the concentrations of the samples being evaluated. The production of NO was evaluated five times for each culture condition and experimental group (five groups).

### 2.9. Quantification of Tumor Necrosis Factor-α

Tumor necrosis factor-α (TNF-α) production, released into the culture medium, was measured using a Human TNF-α Quantikine ELISA Kit (R&D Systems, Minneapolis, MN, USA), following the manufacturer’s instructions. All measurements were conducted in duplicate, employing a quantitative sandwich enzyme immunoassay technique in a 96-well plate format in all the experimental conditions previously mentioned. The optical density of each well was determined at 450 nm using a Cytation3 HT multimodal plate reader (BioTek Instruments Inc., Winooski, VT, USA).

### 2.10. Statistical Analysis

The data are expressed as the mean ± standard deviation (SD) from nine independent experiments, each performed in triplicate unless otherwise stated. Normality testing was conducted using a Shapiro–Wilk test. For numerical variables, a parametric one-way ANOVA test with Tukey’s post hoc test was utilized to identify potential differences between the experimental groups. Statistical significance was determined by *p*-values less than 0.05. Data analysis was performed using GraphPad Prism software version 5.00 for Windows (GraphPad Software, Inc., San Diego, CA, USA).

## 3. Results

### 3.1. Determination of Rhein Working Concentration

**Figure 1:**
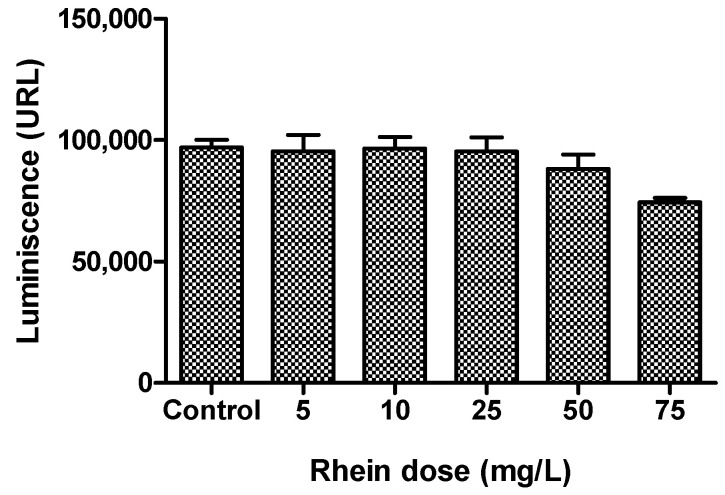
Graphical representation of the dose–response assay to determine the rhein working concentration based on chondrocyte viability.

The average solubility of rhein in human serum samples supplemented with 10% calcium gluconate was 1.8 mg/mL, as determined by the HPLC method. Rhein serum solubility along with previous concentration reported in the literature was considered for the construction of the dose–response assay to determine an optimal working concentration. The dose–response assay revealed that the highest concentration of rhein tested (75 mg/L) reduced chondrocyte viability by more than 20%. The working concentration selected was the highest in which cell proliferation surpassed 90% with respect to the control. The concentration of 50 mg/L was chosen for further experiments (Figure 1).

### 3.2. Effects of Rhein and PRP on Cell Migration

**Figure 2:**
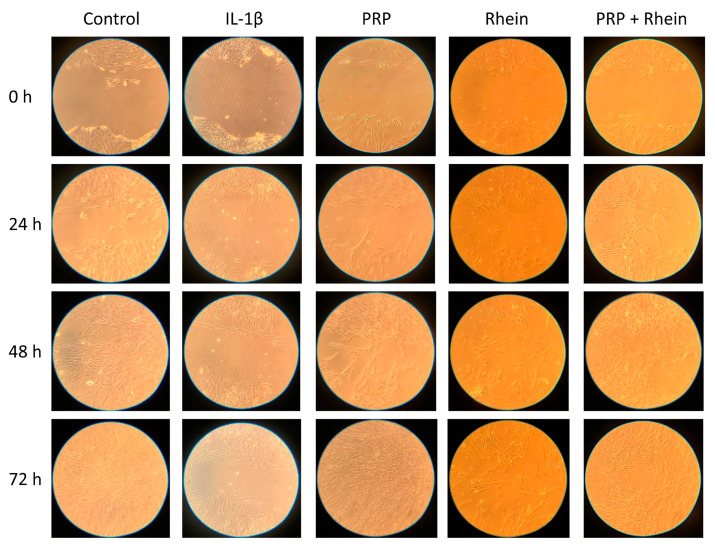
Example of images acquired at 0, 24, 48, and 72 h to assess chondrocyte migration in the wound healing assay under different conditions.

**Figure 3:**
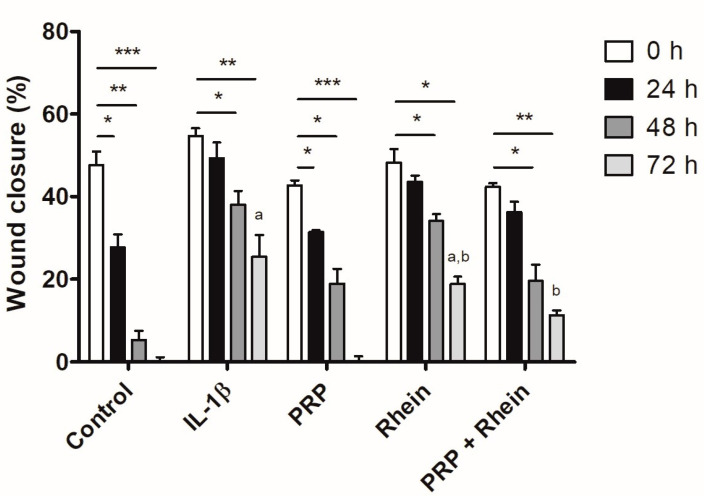
Graphical representation of the gap closure in the wound healing assay for chondrocyte migration. * *p* < 0.05, ** *p* < 0.01, *** *p* < 0.001; ^a^ *p* < 0.05 for IL-1β vs. Rhein, ^b^ *p* < 0.05 for Rhein vs. PRP + Rhein.

The effect of the selected rhein working concentration was evaluated on the migration of human chondrocytes under different conditions, based on the chondrocytes’ capability to fill the wound created in the monolayer culture. The effect of the rhein by itself was not capable of inducing wound closure after 72 h (Figure 2), though the area that remained cell-free was smaller compared to the IL-1β group (rhein 18.8 ± 1.8% vs. IL-1β 25.4 ± 5.4%; *p* < 0.05; Figure 3). The combination of PRP + rhein (11.3 ± 1.2%) induced a greater chondrocyte migration than the rhein alone (rhein 18.8 ± 1.8%; *p* < 0.05; Figure 3), as reflected by the wound area, while the gap could not be entirely filled after 72 h as with control and PRP groups (Figure 2). In all cases, the treatments were able to improve the delay in wound closure caused by IL-1β at 72 h of culture (Figure 3).

### 3.3. Effect of Rhein and PRP on Inflammation- and Matrix Degradation-Related Genes

**Figure 4:**
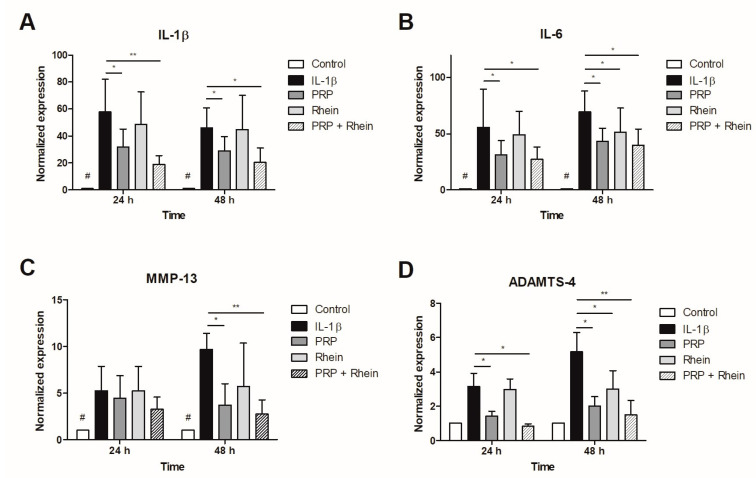
Normalized expression of inflammation− and matrix degradation−related genes in articular human chondrocytes exposed to rhein or PRP after IL-1β stimulation. (**A**) Relative expression of IL-1β. (**B**) Relative expression of IL-6. (**C**) Relative expression of MMP13. (**D**) Relative expression of ADAMTS-5. Data represent the analysis of nine different experiments performed with three replicates. Data are expressed as the mean ± SD. * *p* < 0.05 and ** *p* < 0.01; # *p* < 0.05 vs. all experimental groups. IL-1β, interleukin-1β; IL-6, interleukin 6; MMP-13, matrix metallopeptidase 13; ADAMTS-4, ADAM metallopeptidase with thrombospondin type 1 motif 4.

The normalized gene expression of IL-1β was significantly increased in all experimental groups after incubation with recombinant IL-1β. This effect was counteracted by both PRP alone and PRP + rhein, inducing an mRNA downregulation (Figure 4A). Likewise, IL-6 mRNA levels were upregulated after inflammatory stimulation, and this effect was reversed after PRP and PRP + rhein treatments. At 48 h, the rhein by itself showed a decrease in IL-6 gene expression (Figure 4B). Although IL-1β mRNA levels were lower in the presence of PRP + rhein combination compared to the PRP group, the difference was not statistically significant.

The expression of MMP-13 was significantly influenced by recombinant IL-1β. After 48 h, culture conditions with PRP and the PRP + rhein combination showed a significant decrease in MMP-13 mRNA levels as compared to the inflammation control (Figure 4C). Furthermore, the expression of ADAMTS-4 was similarly downmodulated after the treatment with PRP and PRP + rhein. For this marker, the sole stimulation with rhein also decreased the ADAMTS-4 expression at 48 h (Figure 4D). Despite the combination of PRP + rhein inducing lower expression levels, the difference from the levels of the PRP group was not significant.

### 3.4. Effect of Rhein and PRP on NO Production

**Figure 5:**
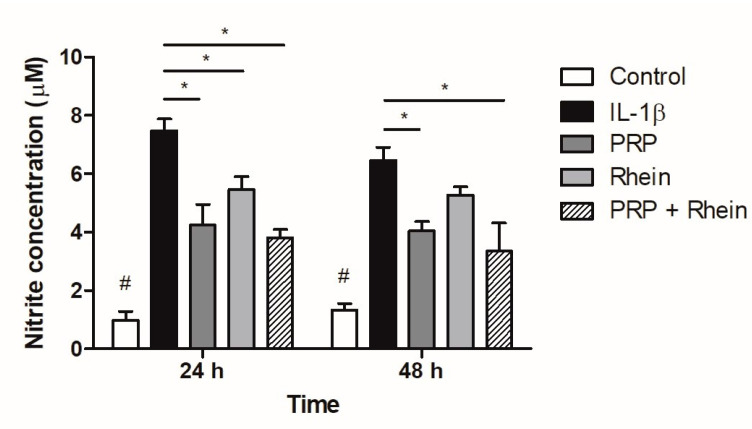
Effect of rhein and PRP on NO production. Data represent the analysis of nine different experiments carried out with five replicates. Data are expressed as mean ± SD. * *p* < 0.05; # *p* < 0.01 vs. all experimental groups. PRP, platelet-rich plasma; NO, nitric oxide.

Nitrite concentrations were determined as an index of NO production, increasing markedly in the groups in which inflammation with IL-1β was induced, compared to the control group at 24 and 48 h (*p* < 0.01; Figure 5). A decrease in the production of NO was especially observed in the groups treated with PRP and the combination of PRP + rhein with respect to the IL-1β group at 24 and 48 h (*p* < 0.05; Figure 5). The effect of rhein alone was not enough to detect a significant reduction after 48 h.

### 3.5. Effect of Rhein and PRP on TNF-α Production

**Figure 6:**
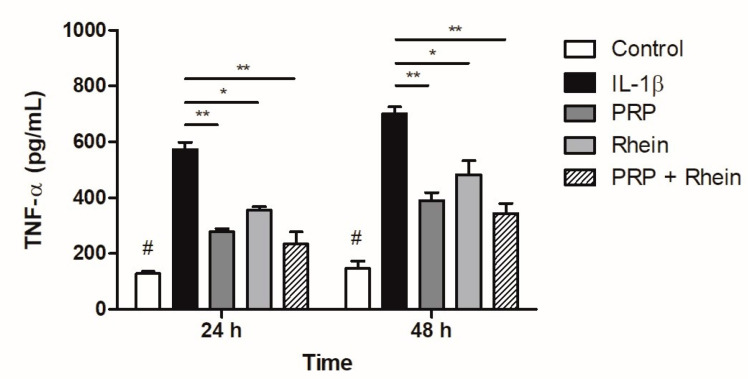
Effect of rhein and PRP on TNF-α production. Data represent the analysis of nine different experiments carried out with three replicates. Data are expressed as the mean ± SD. * *p* < 0.05; ** *p* < 0.01; # *p* < 0.05 vs. all experimental groups. PRP, platelet-rich plasma; TNF-α, tumor necrosis factor-α.

The TNF-α levels produced by chondrocytes stimulated with IL-1β were investigated using an ELISA assay. As shown in Figure 6, the results showed that the concentration of TNF-α in the supernatant increased significantly after IL-1β treatment. However, in the presence of both the PRP and the PRP + rhein, the IL-1β-induced TNF-α production was suppressed at 24 and 48 h (*p* < 0.01).

## 4. Discussion

The main results of this study showed that rhein concentrations lower than 50 mg/L do not affect human chondrocyte viability, and rhein exerts a mild chondroprotective effect by itself. In combination with PRP, no evident synergistic effect was observed; however, in some determinations, this combination produced the greatest reduction in inflammatory and catabolic markers. Due to the poor systemic availability of rhein after diacerein is metabolized, and its gastrointestinal side effects [8], alternative delivery systems for rhein have been studied [25]. We wanted to explore the possibility of combining rhein and PRP as an intra-articular administration alternative. The intra-articular administration of rhein has not been explored before; however, we first wanted to evaluate the chondrocyte response to the rhein + PRP combination.

IL-1β plays a significant role in the development of OA by promoting inflammation and breaking down cartilage. It exerts a strong catabolic influence on cartilage by enhancing the production and function of crucial enzymes involved in the degradation of the extracellular matrix [26]. Therefore, IL-1β can be used to simulate an OA model in vitro in chondrocytes. This was evidenced by the marked increase in TNF-α, NO, IL-1β, IL-6, MMP-13, and ADAMTS-4, as well as a decrease in the chondrocyte migration capacity. The treatments employed in the different experimental conditions were able to counteract or ameliorate the IL-1β-induced changes. Chondrocyte migration was significantly improved in the presence of PRP, which was not surpassed by rhein or PRP + rhein. Evidence on the effect of PRP or rhein specifically on chondrocyte migration is scarce; nevertheless, PRP has been previously shown to stimulate the migration of chondral progenitor cells [27]. In our study, the capability of chondrocytes to fill the area wounded might be directly related to a proliferative effect. In this regard, the proliferative effect of PRP on articular chondrocytes is well documented [28], even when these cells are in the presence of proinflammatory cytokines such as IL-1β [27,29,30]; this is in agreement with our results. On the other hand, rhein did not show the ability to improve gap filling after IL-1β stimulation. Previous studies have shown that rhein can affect chondrocytes’ and synoviocytes’ proliferation in vitro, probably by targeting the cyclin-dependent kinase inhibitor p21, which is active in the cell-cycle regulation of chondrocytes [31,32]. This might help to explain the incomplete wound closure observed after 72 h in the PRP + rhein group.

We further investigated the effect of PRP and rhein on the expression of pro-inflammatory and ECM-related genes. PRP was shown to decrease IL-1β-induced MMP-13, ADAMTS-4, IL-6, and IL-1β mRNA levels. Several reports indicate that PRP has demonstrated the ability to decrease the gene expression of certain proteinases, such as MMP-13, disintegrin, ADAMTS-4, and ADAMTS-5 in animal and human OA chondrocytes [33,34,35,36,37], as well as reducing gene expression of pro-inflammatory markers IL-1β, COX-2, IL-6, IL-8, and IL-18 [34,38,39], thereby reversing the pro-inflammatory and extracellular matrix-degrading effect caused by pro-inflammatory cytokines. Furthermore, rhein has been shown to counteract some of the deleterious effects of IL-1β, including the downregulation of ADAMTS-4, MMP-1, MMP-3, and MMP-13 gene expression in bovine chondrocytes [32]. Moreover, it hinders the expression of genes associated with MMP-1 and MMP-3 (which encode for enzymes involved in cartilage degradation), while promoting the production of aggrecan and collagen, which help maintain cartilage health and prevent joint deterioration [40]. Our data also indicate that rhein was able to reduce ADAMTS-4 and MMP-13 mRNA levels after IL-1β induction. A previous report has shown that inhibition of IL-1β stimulated transcription factors such as NF-κB, which can induce a downregulation of MMPs gene expression in articular chondrocytes [41]. This may help to explain the positive effect on gene expression that we observed. However, rhein did not significantly downmodulate expression of IL-1β and IL-6. Interestingly, the combination of PRP + rhein did not produce an additive effect in modulating the gene expression of the targets evaluated, as the mRNA levels remained with slight decreases with respect to those observed in the PRP group, while maintaining a positive effect. The chondroprotective effect of PRP on chondrocytes exposed to IL-1β has been documented [29,42,43,44].

Rhein exhibits chondroprotective properties by suppressing various substances that contribute to cartilage breakdown, including IL-1β and NO [45,46]. There is a close relationship between these two proinflammatory mediators, since IL-1β has been shown to induce the production of reactive oxygen species such as NO through inducible NO synthase [47]. The induction of this signaling pathway becomes important, because it has been documented that it leads to the inhibition of the synthesis of cartilage ECM [48]. We detected inhibition of NO production after exposure to both rhein and PRP; the combination of PRP + rhein did not have an additive effect on the reduction of NO, reaching levels very similar to those of the PRP group. The inhibition of NO production by rhein and PRP on articular chondrocytes has been reported elsewhere [17,46,49]. Since it was earlier established that NO mediates the stimulatory effect of IL-1β on MMPs synthesis [46,50], it is possible that the effects of rhein and PRP on MMPs’ production result from the inhibition of NO synthesis.

As with NO, the production of TNF-α was also reduced by the effect of both PRP and rhein. This inhibitory effect can be directly related to the counteracting effect of rhein and PRP on IL-1β. Previous research has proposed that the effect of rhein on IL-1β is a result of the inhibition of the NF-κB pathway, resulting in the decrease of transcription factors for different pro-inflammatory mediators such as IL-1β and TNF-α [45,51,52]. The reduction of TNF-α has also been reported in IL-1β articular chondrocytes stimulated by activated PRP, which can be attributed to the bioactive molecules encapsulated in exosomes [53].

Some of the limitations of this study include that some determinations were evaluated within a short period, looking for an immediate effect after an inflammatory stimulation through this first approach. However, since chondrocytes are exposed to chronic inflammatory conditions in knee OA, it would be interesting to determine the activity of PRP + rhein or rhein alone in longer periods. In addition, the effects of PRP and rhein were tested on chondrocytes derived from OA cartilage, instead of chondrocytes derived from healthy tissue. We intended to evaluate the response of chondrocytes under conditions similar to what we expect to find in an OA knee. Both rhein and PRP have been tested in healthy chondrocytes without reporting any kind of harmful effect [29,32,54,55,56]. The selection of the rhein working concentration was based on a dose-dependent viability assay, because we were not certain whether the active metabolite of diacerein would affect the viability of chondrocytes due to the little information available in the literature. An assay of this type evaluating the activity of chondrogenic or inflammatory markers could help to clarify whether a concentration other than the one determined in this study (probably a lower one) would provide a better additive effect for PRP.

## 5. Conclusions

A potential chondroprotective effect of the combination of PRP and rhein was observed in chondrocytes subjected to pro-inflammatory stimulation. Although many of the determinations denoted a better performance of the combination in decreasing the levels of the different targets evaluated, this was not greater enough to detect a significant difference in comparison with PRP treatment alone. It would be convenient to evaluate lower concentrations of rhein with PRP in search of a potential synergistic effect.

## Figures and Tables

**Table 1:** Gene symbols and assay ID of the genes evaluated using RT-qPCR.

Gene	Symbol	Probe Assay ID ^1^
Interleukin-1 β	IL-1β	Hs.PT.58.1518186
Interleukin-6	IL-6	Hs.PT.58.40226675
Matrix metallopeptidase 13	MMP13	Hs.PT.58.40735012
ADAM metallopeptidase with thrombospondin type 1 motif 4	ADAMTS4	Hs.PT.58.4659383
β-2-microglobulin	B2M	Hs.PT.58v.18759587

^1^ Integrated DNA Technologies (IDT), Inc.

## Data Availability

Data are contained within the article.
